# Reliability based geometrically nonlinear bi-directional evolutionary structural optimization of elasto-plastic material

**DOI:** 10.1038/s41598-022-09612-z

**Published:** 2022-04-09

**Authors:** Muayad Habashneh, Majid Movahedi Rad

**Affiliations:** Department of Structural and Geotechnical Engineering, Széchenyi István University, 9026 Gyor, Hungary

**Keywords:** Engineering, Civil engineering

## Abstract

The aim of this paper is to integrate the reliability-based analysis into topology optimization problems. Consequently, reliability-based topology optimization (RBTO) of geometrically nonlinear elasto-plastic models is presented. For purpose of performing (RBTO), the volume fraction is considered reliable since that the application of (RBTO) gives different topology in comparison to the deterministic topology optimization. The effects of changing the prescribed total structural volume constraint for deterministic designs and changing the reliability index for probabilistic designs are considered. Reliability index works as a constraint which is related to reliability condition added into the volume fraction and it is calculated using the Monte-Carlo simulation approach in the case of probabilistic design. In addition, bi-directional evolutionary structural optimization (BESO) method is utilized to study the effect of geometrically nonlinear elasto-plastic design. The plastic behavior can be controlled by defining a limit on the plastic limit load multipliers. The suggested work's efficiency is demonstrated via a 2D benchmark problem. In case of elastic material, a 2D model of U-shape plate is used for probabilistic design of linear and geometrically nonlinear topology optimizations. Furthermore, a 2D elasto-plastic model is considered for reliability-based design to demonstrate that the suggested approach can determine the best topological solution.

## Introduction

Topology optimization of structures aims to achieve the superlative structural performance while fulfilling different constraints. Compared to the other types of structural optimization, topology optimization can substantially be a much freer tool that helps designers in creating novel and exceptionally productive designs for structures. In addition, it can be utilized in cases of discrete structures and continuum structures as well. According to the various scientific research, it can be remarked that despite of the existence challenges of structural topology optimization, it tends to be one of the most remunerating economically techniques.

Recently, topology optimization has turned into a very dynamic area of research and enhancement. Therefore, various topology optimization methods have reached the level of practical applications. Such as solid isotropic material with penalization (SIMP) method^1,2^ where the material density is considered as the design variable in this approach. Another topology optimization approach is called the level-set method which was proposed by Sethian^3^, the essential concept relies on the idea of moving material interface which is controlled by Hamilton–Jacobi partial differential equation. In comparison with different topology optimization approaches, the moving morphable components (MMC) method^4,5^ can achieve the optimization process in geometrical and an explicit way. Evolutionary structural optimization (ESO) method^6^ is one of the developed methods in structural optimization which has been undergone massive enhancement since its first proposition. The fundamental concept of (ESO) is that the incompetent materials are removed step by step, therefore the optimal structure can be achieved. However, (ESO) method revealed some designs issues related to its application. To overcome these insufficiencies, (ESO) developed to be bi-directional evolutionary structural optimization (BESO). Generally, (BESO) method counts on the idea of removing solid elements with smallest sensitivity numbers and switch the voids elements with the greatest sensitivity numbers into solid elements simultaneously within the prescribed design domain for each iteration.

The most examined design case is that considering structural mean compliance minimization, subjected to a volume constraint^7^. Moreover, the latest versions of BESO method have effectively shown a promising performance when considering it for different topology design problems such as geometrically nonlinear^8,9^, composite materials^10,11^, and elasto-plastic analysis^12^.

Most of the existence research work, deal with linear finite element topology optimization problems. However, a very limited research works deal with the geometrically nonlinear topology optimization. Despite the fact that the linear assumptions are not always adequate, for instance, in the cases of compliant and energy absorption as well as fracture failure designs^13–18^. In the study of Chen et al.^19^, a geometrically nonlinear topology optimization formulation considering maximum displacement of nodes as a constraint was introduced. Zhu et al.^20^ presented a code of SIMP topology optimization method by considering geometrically nonlinear problems.

Dealing with uncertainties in case of structural topology optimization is crucial since the designer must deal with it, especially in cases of applied loads variations of the material properties and geometrical dimensions. Thus, reliability-based topology optimization is gaining considerable attention to make it more practical^21,22^. Reliability-based design optimization (RBDO) was considered in the study of Meng et al.^23^, where a transformation approach to solve the complexity of high-fidelity engineering systems by converting an arbitrary high-fidelity RBDO into classical arbitrary low-fidelity RBDO. A robust topology optimization approach by considering randomly loading magnitude and direction for fiber-reinforced plastic materials was proposed in the study of Xu et al.^24^. Also, by considering uncertainty in the magnitude and the direction of the applied load, Dunning et al.^25^ presented a robust topology optimization approach considering the minimization of the mean compliance. For purposes of handling epistemological and random uncertainties in topology optimization, Meng et al.^26^ proposed hyprid reliability-based topology technique depending on fuzzy and probabilistic theory to illustrate the uncertainties multi-source. Cheng and Guo^27^ proposed a so-called ε-relaxed technique for structural topology optimization problems of discrete structures. A probabilistic optimal topology design problem was studied by Lógó^28^ for continuum type of structures where the location of the applied loads are given randomly. Meng et al.^29^ presented a robust topology optimization method to simultaneously resolve the uncertainties based on the probabilistic methodologies. A probabilistic model was introduced to topology optimization problems by assigning reliability constraints into deterministic problems by Kharmanda et al.^30^.

When the material or part of it undergoes elasto-plastic deformation, its properties will be changed according to the level of plastic strain. Thus, it will be resulted in a nonlinear relation between stress and strain. Accordingly, this will affect the strain energy which is considered important since the major of structural engineering problems considering minimizing it^31,32^.

Li et al.^33^ proposed a multi-material topology optimization which can conform multiple plasticity as well as multiple hardening models in composite structures. Linearly elastic and perfectly plastic material models of structural topology optimization have been investigated by^34^ to find the optimum solution of a structure according to a given load. Boissier et al.^35^ investigated the influence of elasto-plastic shakedown constraints by integrated into structural topology optimization framework. Recently elasto-plastic limit analysis of reliability-based geometrically nonlinear topology optimization was proposed in the study of Rad et al.^36^. In this research, (BESO) method was used to consider the effect of geometrically nonlinear design for elasto-plastic analysis. Plastic behavior also controlled by considering a bound on the plastic limit load multipliers using limit analysis.

This study is considering the effect of changing the prescribed total structural volume constraint for the deterministic topology optimization design as well as the effect of changing the reliability index constraint in case of probabilistic topological design. Thus, this paper presents reliability-based geometrically nonlinear BESO method of elasto-plastic material. Considering that the geometrically nonlinear behavior was modelled by using total Lagrangian finite element formulation. In addition, elasto-plastic materials have been used in order to investigate their effect on topology optimization problems. The reason of considering the volume fraction as random variable that the application of reliability-based topology optimization shows different topological results comparing to those which are obtained through deterministic designs. Besides, the Monte-Carlo sampling technique has been applied to calculate failure probability. In order to perform elasto-plastic analysis, plastic limit analysis is considered. 2D elastic and 2D elasto-plastic numerical examples are introduced to examine the effectiveness of the proposed method. Also, a comparison between deterministic and probabilistic design is introduced.

The rest of the paper is coordinated as follows: “Topology optimization: deterministic problem” section gives an outline of deterministic topology optimization problem. “Introducing probabilistic design into topology optimization” section demonstrates the probabilistic design of BESO topology optimization. The newly improved BESO procedure is presented in “The newly improved BESO procedure” section. Numerical examples are presented in “Numerical examples” section. Finally, the conclusion, clarifications and future prospect are given in “Conclusions” section.

## Topology optimization: deterministic problem

### Geometrically nonlinear analysis

For nonlinear large displacements analysis, total nonlinear Lagrangian finite element model has been used as:

1 $${\eta }_{ij}=\frac{1}{2}\left({u}_{i,j}+{u}_{j,i}+{u}_{k,i} {u}_{k,j}\right)$$

where $u$ represents the point-wise displacement, and the subscripts refer to the coordinate axes.

2 $$d\eta =B\left(U\right)dU$$

where $B$ refers to the finite element matrix which transforms the displacement change $dU$ into a strain changing, and $U$ is the vector of finite element displacement. The Hooke's law for intermediate material densities can be expressed as:

3 $${s}_{ij}= {({p}_{e})}^{p} {C}_{ijkl}^{0} {\eta }_{kl}$$

where ${s}_{ij}$ is the component of second Piola–Kirchhoff stress, ${p}_{e}$ stands for Péclet number, $p$ is the penalization power, ${C}_{ijkl}^{0}$ represents the constitutive tensor of solid isotropic material, ${\eta }_{kl}$ is the Green–Lagrange strains. Thus, the residual can be defined as the error of the obtained equilibrium.

4 $$R\left(U\right)= P - {\int }_{V}{B}^{T}\mathrm{ s }dV.$$

where $s$ refers to the vector of Piola–Kirchhoff stress and $P$ represents the applied force. The equilibrium found when the residual vector equal to zero vector. As a rule, the finite element equilibrium $\left({4}\right)$ is solved by using Newton–Raphson iterative scheme.

5 $${K}_{T}= - \frac{\partial R}{\partial U}.$$

where ${{\varvec{K}}}_{{\varvec{T}}}$ represents the tangential stiffness matrix.

### Introducing elasto-plastic analysis into BESO method

One can find different scientific research papers and books which explaining BESO method in details. However, a brief description with the proposed extensions of BESO method is discussed here in this paper.

The aim of topology optimization for the most cases is to find the best system’s performance while satisfying a specified volume constraint. In BESO method, the structure can reach the optimal solution by removing and adding the elements simultaneously^37^.

Thus, the formulation of topology optimization problem considering volume constraint can be outlined as:

6.a $$Min. C= {u}^{T}Ku$$

6.b $$Subjected to: {V}^{*} - \sum_{i=1}^{N}{V}_{i}{x}_{i}= 0$$

6.c $${x}_{i}\in \left\{\mathrm{0,1}\right\}$$

6.d $$\frac{{V}^{*}}{{V}_{0}}-{V}_{f}\le 0.$$

where $C$ stands for the mean compliance, $K$ refers to global stiffness matrix and $u$ represents displacement vectors. On the other hand, ${V}_{i}$ represents elemental volume, ${V}^{*}$ is the volume of structure, and $N$ refers to the number of elements within design domain. Considering that ${x}_{i}$ is a binary variable which is equal to $(1)$ when the element exists while it equals to $(0)$ in case of absence. ${V}_{0}$ is the design domain volume and ${V}_{f}$ represents volume fraction ratio. By definition of the sensitivity number of $\text{i-th}$ element and The change in the elastic strain energy is expressed as follows:

7 $${\alpha }_{i}^{e}=\Delta {C}_{i}= \frac{1}{2}{\left\{{u}_{i}\right\}}^{T}\left[{K}_{i}\right]\left\{{u}_{i}\right\}.$$

where ${u}_{i}$ represents the nodal displacement, and ${K}_{i}$ indicates the stiffness matrix of the $\text{i-th}$ element. Taking into consideration that initial sensitivity numbers of void elements equal to zero. The occurrence of checkerboard pattern or mesh-dependency problem causes difficulty in reaching the ‘optimal’ case of structure. To overcome checkerboard pattern which might cause difficulty for reaching optimum solution, filter scheme is applied in BESO method since it is used to acquire the sensitivity number of void elements^7^.

The constant length scale of the filter scheme ($${r}_{min})$$ is used to differentiate the nodes which affect the $$\text{i-th}$$ element sensitivity. The nodes which are located within the sub-domain $${\Omega }_{i}$$ assist in the computation of $$\text{i-th}$$ element improved sensitivity number as follows:

8 $${\alpha }_{i}=\frac{\sum_{j=1}^{K}w({{r}_{ij})\alpha }_{j}^{n}}{\sum_{j=1}^{K}w({r}_{ij})}.$$

where ${\text{k}}$ represents the number of nodes within ${\Omega }_{i}$, $w({r}_{ij})$ is the factor of linear weight which expressed as:

9 $$w\left({r}_{ij}\right)= {r}_{\mathrm{min}- {r}_{ij}}$$

To define the nodal sensitivity numbers, the following formulation is used:

10 $${\alpha }_{j}^{n}=\sum_{i=1}^{M}{w}_{i}{\alpha }_{j}^{e}$$

where ${\text{M}}$ is the number of connected elements to th*e* $\text{j-th}$ node, and ${w}_{i}$ is $\text{i-th}$ element weight factor. Consequently, ${w}_{i}$ can be determined by:

11 $${w}_{i}=\frac{1}{M-1}\left(1-\frac{{r}_{ij}}{\sum_{i=1}^{M}{r}_{ij}}\right)$$

where ${r}_{ij}$ stands for the distance between the center *of* $\text{i-th}$ element and the jth node. For purposes of stabilizing the process of evolution, Huang et al.^38^ tracked down a powerful technique by implementing averaging scheme which can be expressed as:

12 $${\alpha }_{i}=\frac{{\alpha }_{i}^{k}+{\alpha }_{i}^{k-1}}{2}$$

where ${\text{k}}$ indicates the current iteration number. The target volume for next iteration ${V}_{k+1}$ should be considered first by the following equation:

13 $${V}_{k+1}={V}_{k}(1\pm ER)$$

where ${\text{ER}}$ and ${\text{k}}$ are evolutionary ratio and the current iteration number, respectively. After satisfying volume constraint, the volume of the structure will be constant for the rest iterations based on the following formulation:

14 $${V}_{k+1}={ V}^{*}.$$

Then, the elements are sorted based on their number values in descending way. For solid elements, it will be eliminated if:

15 $${\alpha }_{i}\le {\alpha }_{del}^{th}$$

while for void elements, it will be added if:

16 $${\alpha }_{i}>{\alpha }_{add}^{th}$$

where ${\alpha }_{del}^{th}$ stands for threshold sensitivity numbers of erasing and ${\alpha }_{add}^{th}$ is the threshold sensitivity numbers of adding elements.

The optimization process will constantly perform by deleting and adding elements until the constraints ($6.b$) to ($6.d$) are satisfied as well as the following convergence criteria is satisfied:

17 $$error=\frac{|\sum_{i=1}^{N}({F}_{k-i+1}-{ F}_{k-N-i+1} )|}{\sum_{i=1}^{N}{F}_{k-i+1}}\le \tau$$

where $N$ is standing for integer number which resulted in a stable compliance for at least ten successive iterations, $F$ is the goal function,$\tau$ is convergence tolerance, and $k$ is the number of the current iteration.

The newly improved method depends on the idea of elasto-plastic limit analysis. To understand the basic idea of the newly developed method one has to repeat the fundamental formulation and proof of the static principle of limit analysis. The limit analysis can be demonstrated as follows: assume an elasto-plastic body exposed to a given force ${F}_{i}$ and consider that this force is increasing consistently. Thus, the proportional load can be determined as:

18 $${F}_{i}=m {F}_{0}$$

where ${F}_{0}$ is the predefined external applied forces while $m$ is a monotonically expanding scalar boundary which is called the load multiplier. As much $m$ increases, as much the plastic regions appear in the design domain until characterized intensity ${m}_{p}$ is reached thus unrestricted state of plastic flow will be reached. The state where the elasto-plastic body or parts of it go through unrestricted plastic deformation under consistent external load is known as the plastic limit state. The relating load multiplier ${m}_{p}$ indicates the plastic limit load multiplier. Thus, the plastic limit load is formulated as:

19 $${F}_{p}={m}_{p}{F}_{0}$$

Taking into consideration that the load multiplier cannot exceed the plastic-limit load multiplier. At plastic limit state, stresses and external forces can keep up the body’s static equilibrium. Consequently, the equilibrium equations will be applied. By considering the stress ${\sigma }_{ij}$ within a body which ensures quasi-static equilibrium with plastic limit load, and the arbitrary statically admissible stress ${\sigma }_{ij}^{s}$ and force ${F}_{is}={m}_{s}{F}_{0}$ which satisfy the yield conditions.

20 $$f({\sigma }_{ij}^{s},k)\le 0$$

where $k$ stands for material’s plastic properties. By introducing the strain rate ${\dot{\varepsilon }}_{ij}$ and velocities ${v}_{i}$ considering a deformable body with volume $V$ and loading surface ${S}_{q}$, the principle of virtual velocities can be applied for both stress and force fields.

21 $$\underset{V}{\overset{}{\int }}{\sigma }_{ij}{\dot{\varepsilon }}_{ij}dV={m}_{p}\underset{{S}_{q}}{\overset{}{\int }}{F}_{0}{v}_{i}dS$$

22 $$\underset{V}{\overset{}{\int }}{\sigma }_{ij}^{s}{\dot{\varepsilon }}_{ij}dV={m}_{s}\underset{{S}_{q}}{\overset{}{\int }}{F}_{0}{v}_{i}dS$$

By subtracting two equations yields:

23 $$\underset{V}{\overset{}{\int }}{({\sigma }_{ij}-\sigma }_{ij}^{s}){\dot{\varepsilon }}_{ij}dV=({m}_{p}-{m}_{s})\underset{{S}_{q}}{\overset{}{\int }}{F}_{0}{v}_{i}dS$$

Thus, referring to the convexity of yield surface and normality rule:

24 $${({\sigma }_{ij}-\sigma }_{ij}^{s}){\dot{\varepsilon }}_{ij}\ge 0$$

Consequently, Eq. ($23$) yields:

25 $$({m}_{p}-{m}_{s})\underset{{S}_{q}}{\overset{}{\int }}{F}_{0}{v}_{i}dS\ge 0$$

In this formulation, the integral shows the work done according to the external forces on the actual velocities of the body. According to the definition of plastic limit state, the work cannot be negative, thus, ${m}_{s}-{m}_{p}\le 0$.

Considering previously mentioned steps in this section, this part can be added to the topology optimization problem, therefore, the method won’t have more complicated nature mathematically.

Accordingly, the optimization problem subjected to plastic limit constraint can be expressed as:

26.a $$Minimize: C= {u}^{T}Ku$$

26.b $$Subject to: {V}^{*} - \sum_{i=1}^{N}{V}_{i}{x}_{i} = 0$$

26.c $${x}_{i}\in \{\mathrm{0,1}\}$$

26.d $${m}_{s}-{m}_{p}\le 0.$$

Here Eqs. ($26.a-c$) play similar parts as Eqs. ($6.a-c$). While Eq. ($26.d$) shows the adoption of plastic limit multiplier.

## Introducing probabilistic design into topology optimization

By calling the basic idea of reliability analysis and assume ${X}_{R}$ stands for the non-negative limit of ${X}_{S}$, the failure issue then can be illustrated by ${X}_{R}\le {X}_{S}$. Let ${X}_{R}$ and ${X}_{S}$ be independent random variables having probability density functions ${f}_{R} ({X}_{R})$ and ${f}_{S} ({X}_{S})$. The failure probability can be estimated by:

27 $${P}_{f}= P\left[{X}_{R}\le {X}_{S}\right]= {\iint }_{{X}_{R}\le {X}_{S}}{f}_{R} \left({X}_{R}\right){f}_{S} \left({X}_{S}\right)d{X}_{R}d{X}_{S}.$$

The above problem can be defined in terms of the so-called bound state function which is described by:

28 $$g\left({X}_{R},{X}_{S}\right)={X}_{R}-{X}_{S}.$$

Taking into consideration that $g \le 0$ indicates the domain of failure ${D}_{f}$. Thus, the failure probability ${P}_{f}$ is formulated by:

29 $${P}_{f}={F}_{g}\left(0\right).$$

Furthermore, ${P}_{f}$ can be determined according to:

30 $${P}_{f}={\int }_{g({X}_{R},{X}_{S})\le 0}f\left(X\right)dX={\int }_{{D}_{f}}f\left(X\right)dX.$$

The Monte-Carlo sampling technique is used in this study to find the reliability index $(\beta )$ by determining probability of failure $({P}_{f})$. In general, the concept of Monte-Carlo method relies on the idea of generating realizations $x$ of the random vector $X$ from the probability density function ${f}_{X}(x)$. Consequently, failure probability can be determined by calculating the total number of points in the failure domain with respect to the number of generated points^39,40^. This idea can be expressed by presenting an indicator function of the failure domain ${D}_{f}$:

31 $$\chi _{{D_{f} }} \left( x \right) = \left\{ {\begin{array}{*{20}c} {1\quad if\quad x \in D_{f} } \\ {0\quad if\quad x \notin D_{f} } \\ \end{array} } \right\}$$

Then writing the probability of failure (30) as:

32 $${P}_{f}={\int }_{-\infty }^{+\infty } . . . {\int }_{-\infty }^{+\infty }{\chi }_{{D}_{f}}\left(x\right){f}_{X}\left(x\right)dx.$$

${\chi }_{{D}_{f}}\left(X\right)$ is consequently a random variable along two points distribution.

33 $${\mathbb{P}}\left[ {\chi }_{{D}_{f}}\left(X\right)=1\right]= {P}_{f}$$

34 $${\mathbb{P}}\left[ {\chi }_{{D}_{f}}\left(X\right)=0\right]= {1-P}_{f}$$

where ${P}_{f}={\mathbb{P}}[X\in {D}_{f }]$. The mean value and variance of ${\chi }_{{D}_{f}}\left(X\right)$ are characterized by:

35 $${\mathbb{E}}\left[{\chi }_{{D}_{f}}\left(X\right)\right]=1\cdot {P}_{f}+0\cdot \left(1-{P}_{f}\right)={P}_{f}$$

36 $${\mathbb{V}}ar\left[{\chi }_{{D}_{f}}\left(X\right)\right]={\mathbb{E}}\left[{\chi }_{{D}_{f}}^{2}\left(X\right)\right]- \left( {{\mathbb{E}}\left[ {\chi _{{D_{f} }} \left( X \right)} \right]} \right)^{2} ={P}_{f}-{P}_{f}^{2}={P}_{f}\left(1-{P}_{f}\right).$$

To determine ${P}_{f}$ by using Monte-Carlo technique, the following mean value estimator is used:

37 $$\widehat{\mathbb{E}}\left[{\chi }_{{D}_{f}}\left(X\right)\right]=\frac{1}{Z}\sum_{z=1}^{Z}{\chi }_{{D}_{f}}({X}^{(z)})={\widehat{P}}_{f}$$

where ${X}^{(z)}$ are independent random vectors ($z=1,\dots ,Z$) having probability density functions given by ${f}_{X}(x)$. Due to the presence of uncertainties, volume fraction ${V}_{f}$ is considered as a random variable in case of probabilistic designs with mean value and standard deviation and it follows the Gaussian distribution having mean ${\mathbb{E}}$ and variance ${\mathbb{V}}ar$. Then, the mean and the variance of the estimator can be simply computed as following:

38 $${\mathbb{E}}\left[{\widehat{P}}_{f}\right]=\frac{1}{Z}\sum_{z=1}^{Z}{\mathbb{E}}\left[{\chi }_{{D}_{f}}\left({X}^{\left(z\right)}\right)\right]=\frac{1}{Z}Z{P}_{f}={P}_{f}$$

39 $${\mathbb{V}}ar\left[{\widehat{P}}_{f}\right]=\frac{1}{{Z}^{2}}\sum_{z=1}^{Z}{\mathbb{V}}ar\left[{\chi }_{{D}_{f}}\left({X}^{\left(z\right)}\right)\right]=\frac{1}{{Z}^{2}}Z{P}_{f}\left(1-{P}_{f}\right)=\frac{1}{Z}{P}_{f}\left(1-{P}_{f}\right).$$

Then, by formulating the reliability index $(\beta )$ the reliability constraint can be introduced as:

40 $${\beta }_{target}-{\beta }_{calc}\le 0$$

where ${\beta }_{calc}$ is the calculated reliability index for each iteration and when it reaches the target value of reliability index ${\beta }_{target}$, the program will be terminated since that this constraint is satisfied.

To calculate ${\beta }_{target}$ and ${\beta }_{calc}$, the following expressions are used:

41 $${\beta }_{target}=-{\Phi }^{-1}\left({P}_{f,target}\right);$$

42 $${\beta }_{calc}=-{\Phi }^{-1}\left({P}_{f,calc}\right).$$

where ${\Phi }^{-1}$ is the inverse of the standard normal cumulative distribution function.

Thus, the probabilistic topology problem can be formulated as:

43.a $$Minimize: C= {u}^{T}Ku$$

43.b $$Subject \,to: {V}^{*} - \sum_{i=1}^{N}{V}_{i}{x}_{i} = 0$$

43.c $${x}_{i}\in \left\{\mathrm{0,1}\right\}$$

43.d $${\beta }_{target}-{\beta }_{calc}\le 0$$

Here Eqs. ($43.a-c$) have same roles as Eqs. ($6.a-c$). While Eq. ($43.d$) represents the added constraint which is related to reliability condition on the volume fraction.

## The newly improved BESO procedure

Overall, the topology optimization design gives a non-convex problem with non-unique solutions. It is acknowledged that the plasticity’s extremum theorems lead to convex problems thus the solutions are novel. After this brief argument of the extended mathematical nature of the problem, the probabilistic elasto-plastic optimization problem therefore can be formulated by introducing reliability and plastic limit constraints as the following:

44.a $$Minimize: C= {u}^{T}Ku$$

44.b $$Subject\, to: {V}^{*} - \sum_{i=1}^{N}{V}_{i}{x}_{i} = 0$$

44.c $${\beta }_{target}-{\beta }_{calc}\le 0$$

44.d $${x}_{i}\in \{\mathrm{0,1}\}$$

44.e $${m}_{s}-{m}_{p}\le 0.$$

**Figure 1:**
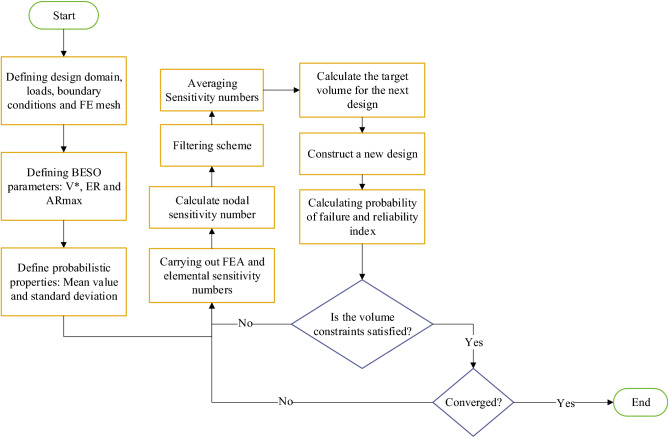
Flow chart of the elasto-plastic reliability-based BESO topology algorithm.

Figure 1 represents the flow chart of the elasto-plastic reliability-based BESO topology optimization algorithm, the procedure is summarized as follows:

1. Defining the model including load and boundary conditions.
2. Carrying out FEA then calculate the sensitivity numbers.
3. Applying the averaging sensitivity number technique.
4. Specify the target volume value for the next iteration.
5. Introducing and eliminating of elements.
6. Repeat steps from 2 to 5 until the defined constraints (${\beta }_{target}-{\beta }_{calc}\le 0$ , ${m}_{s}-{m}_{p}\le 0,$ and ${V}^{*} - \sum_{i=1}^{N}{V}_{i}{x}_{i} = 0$, $\frac{{V}^{*}}{{V}_{0}}-{V}_{f}\le 0$) are satisfied as well as the convergence criteria is satisfied.

## Numerical examples

In this section, three numerical examples are studied. Two of them are considered for elastic geometrically nonlinear model. 2D plate supported at all corners is considered as first example, for the purpose of investigation the efficiency of the presented work, the results are compared with a benchmark problem of Fernandes et al.^41^. Second example is a 2D U-shaped plate problem for geometrically nonlinear elastic probabilistic analysis. Finally, a 2D U-shaped plate problem is considered as third example for geometrically nonlinear elasto-plastic probabilistic analysis. For purposes of evaluating failure probability, Monte-Carlo simulation is considered. Also, volume fraction ${V}_{f}$ is considered random to characterize probabilistic nature for reliability assessment.

### 2D elastic problem: simply supported plate

**Figure 2:**
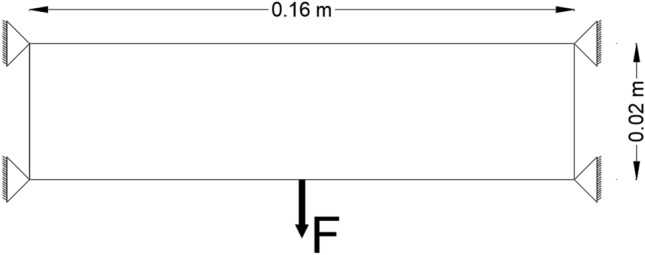
Simply supported plate benchmark problem.

A topology optimization problem considering stiffness maximization of slender plate which is simply supported from all corners is considered as the first problem in this study. Figure 2 illustrates this optimization problem. $F=30 N$ is acting at the bottom edge’s center. The design is $0.001$ m in thickness. The volume fraction values of the available material are set to be 47%, 43% and 40% of the design domain. While the used material has Young’s modulus of 30 MP and Poisson’s ratio of 0.3. BESO parameters are $ER=17.5\%$, ${AR}_{max }=1\%$ , ${r}_{min }= 0.003\,m$ and $\tau =1\%$. ${V}_{f}$ has probabilistic properties which are 4 $0\%$ and $5\%$ for mean value and variance, respectively. The number of sample points is assumed ( $Z=3.0\times {10}^{6})$.

To exhibit the validation of the proposed method, the topological optimized shapes of deterministic linear designs as well as for geometrically nonlinear designs were compared with the benchmark problem which had been done by Fernandes et al.^41^. The results of deterministic design were obtained considering different values of ${V}_{f}$. In addition, in case of probabilistic design, the obtained results shown by considering different values of ${\beta }_{target}$.

**Figure 3:**
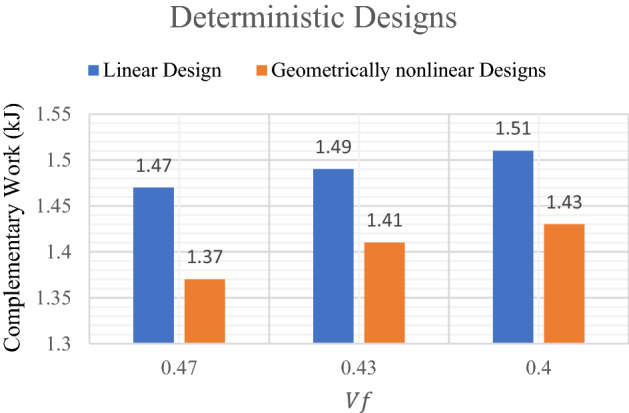
Complementary work values of deterministic design of the model.

**Figure 4:**
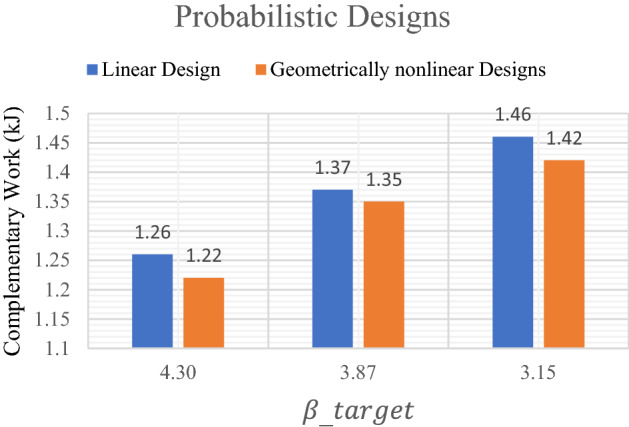
Complementary work values of probabilistic design of the model.

A comparison between linear and geometrically nonlinear analysis of the results of deterministic designs of the optimized shapes and complementary work by considering different values of volume fraction. Similarly, comparison between probabilistic linear and geometrically nonlinear analysis are considered in this example. Taking into consideration that the displacement values are considered according to complementary work for all considered models based on the suggestion of^42^ as it can be seen in different bar charts figures later (Figs. 3, 4).

The results which are presented in Table 1 represent the optimal solution of deterministic linear design of the model while Table 2 shows the results of deterministic geometrically nonlinear analysis.

Other comparisons according to the obtained complementary work are shown in Fig. 3. It can be noted that the complementary work value for geometrically nonlinear design is smaller than that for linear design for the same value of ${V}_{f}$. Also, from Fig. 3 It can be noticed that for ${V}_{f}=0.47$, the complementary work has been decreased by $6.80\mathrm{\%}$ from $1.47$ kJ in case of linear design to $1.37$ kJ in case of geometrically nonlinear design. While for the ${V}_{f}=0.40$, the complementary work has been reduced by $5.30\mathrm{\%}$ from $1.51$ kJ to $1.43$ kJ.

It is worth to mention that as ${V}_{f}$ decreases the complementary work increases in case of deterministic analysis for elastic linear and geometrically nonlinear designs.

Table 3 represent the optimal solution of probabilistic linear design of the slender plate problem. On the other hand, Table 4 shows the results of probabilistic geometrically nonlinear design. As a result, the application of RBTO shows different topological results comparing to those which are obtained in case of deterministic designs.

Similar to the comparisons of the deterministic results, here a comparison between results of probabilistic linear and geometrically nonlinear designs are presented in Fig. 4. It can be noted that the complementary work value for linear design is smaller than that for geometrically nonlinear design for the same value of ${\beta }_{target}$. It can be also noticed from Fig. 4 that for ${\beta }_{target}=4.30$, the complementary work has been decreased by $3.17\mathrm{\%}$ from $1.26$ kJ in case of linear design to $1.22 kJ$ in case of geometrically nonlinear design. Besides, for the ${\beta }_{target}=3.15$, the complementary work has been reduced by $2.74\mathrm{\%}$ from $1.46$ kJ to $1.42$ kJ.

The obtained results which are shown in Fig. 4 indicates that the complementary work increases as ${\beta }_{target}$ decreases in case of probabilistic design for both linear and geometrically nonlinear analysis.

Besides, it is worth to mention that when introducing ${\beta }_{target}$ constraint, the complementary work has been effectively decreased for elastic linear and geometrically nonlinear designs form those results which have been obtained from deterministic designs.

Finally, since.

**Table 1:**
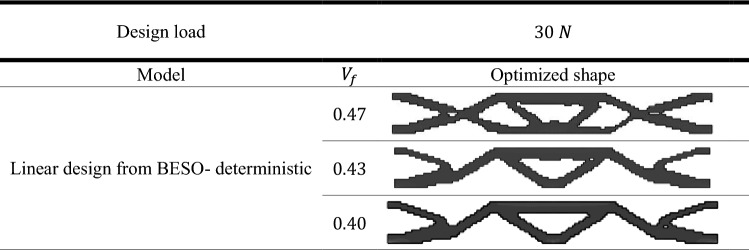
The optimal solution of deterministic linear design of the model.

**Table 2:**
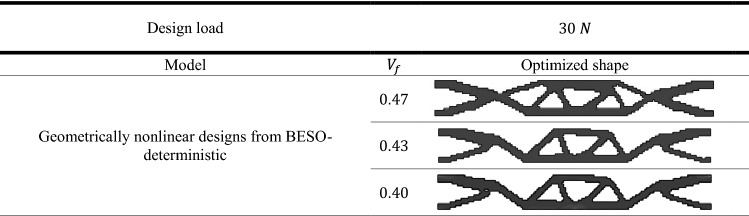
The optimal solution of deterministic geometrically nonlinear design of the model.

**Table 3:**
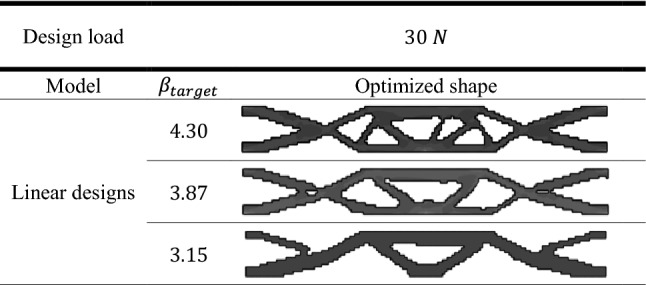
The optimal solution of probabilistic linear design of the model.

**Table 4:**
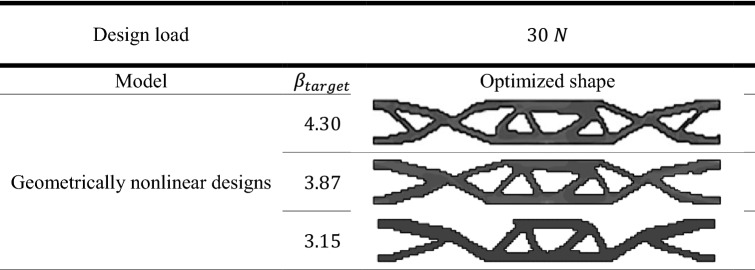
The optimal solution of probabilistic geometrically nonlinear design of the model.

### 2D elastic problem: U-shaped plate

**Figure 5:**
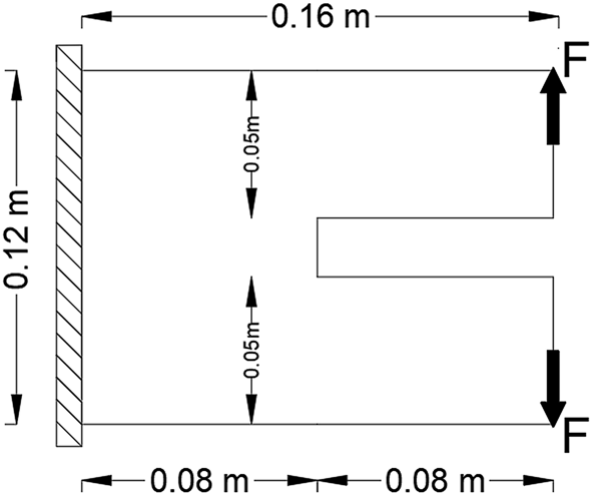
2D U-shaped plate model.

The second example in this paper is considering linear and geometrically nonlinear elastic analysis for probabilistic design of 2D U-shaped plate problem as it shown in Fig. 5. Similar to what mentioned in previous example, The Monte-Carlo sampling technique is considered and for probabilistic analysis. Besides, ${V}_{f}$ is introduced as a random variable to exhibit the probabilistic nature. The material properties are Young’s modulus of $70 GPa$ and Poisson’s ratio of $0.3$. Plate with thickness $0.010$ m is assumed $.$ The BESO parameters are ${AR}_{max }=1\mathrm{\%},$ $ER=1\mathrm{\%}$, ${r}_{min }= 0.006$ m and $\tau =1\mathrm{\%}$. Three different ${V}_{f}$ are considered in this problem $.$ The initial predefined applied load is equal to $F=12.5$ kN. For probabilistic analysis, mean value of $60\mathrm{\%}$ and variance of $5\mathrm{\%}$ are assumed for the random variable ${V}_{f}.$ In addition, for ${P}_{f}$ calculation purposes, $Z=3.0\times {10}^{6}$ is considered as the number of generated points.

A comparison between linear and geometrically nonlinear analysis of the results of deterministic designs of the optimized shapes, complementary work and maximal Huber-Mises-Hencky stress by considering three different values of volume fraction. Also, a comparison between linear and geometrically nonlinear analysis of probabilistic designs is considered in this example.

**Table 5:**
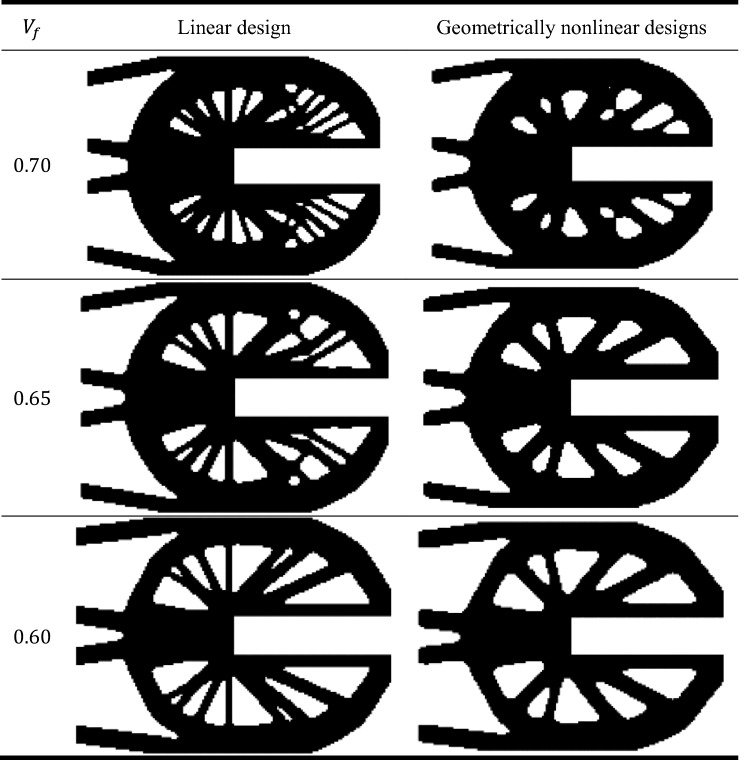
Topological resulted shapes of the U-shaped plate in case of deterministic design.

**Figure 6:**
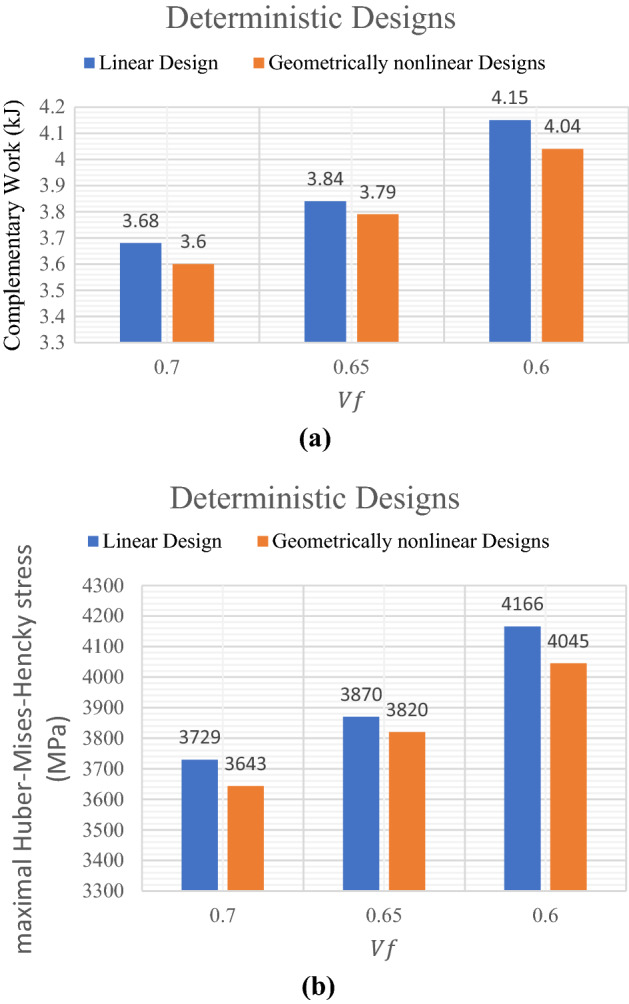
Comparison between linear and geometrically nonlinear deterministic design by considering: (**a**) complementary work, (**b**) maximal Huber-Mises-Hencky stress.

The results shown in Table 5 represent the optimal topological shapes of linear design and geometrically nonlinear design of the U-shaped plate. While Fig. 6 represents a comparison between the results of deterministic design of linear and geometrically nonlinear topology optimization according to the obtained complementary work and mean stresses considering different values of ${V}_{f}$.

Similar to previous problem, the displacement values are considered according to the complementary work for all considered models. From Fig. 6a we can notice that the complementary work has been decreased by $2.17\%$ from $3.68$ kJ in case of linear design to $3.60$ kJ in case of geometrically nonlinear design when ${V}_{f}=0.70$. Also, for the ${V}_{f}=0.60$, the complementary work has been reduced by $2.65\%$ from $4.15$ kJ to $4.04$ kJ. On the other hand, according to Fig. 6b, the maximal Huber-Mises-Hencky stress has been decreased by $2.30\%$ from $3729$ MPa in case of linear design to $3643$ MPa in case of geometrically nonlinear design when ${V}_{f}=0.70$. Also, for the ${V}_{f}=0.60$, the maximal Huber-Mises-Hencky stress has been reduced by $2.90\%$ from $4166$ MPa to $4045$ MPa.

It can be noted that the complementary work value for geometrically nonlinear design is smaller than that for linear design for the same value of ${V}_{f}$. Also, it can be also noticed from Fig. 6 that in general for both linear and geometrically nonlinear designs, as ${V}_{f}$ decreases the complementary work increases. Besides, according to another comparison which is shown in Fig. 6b, we can say that as ${V}_{f}$ decreases the maximal Huber-Mises-Hencky stress increases too for both linear and geometrically nonlinear designs.

Table 6 represent the optimal solution of linear and probabilistic geometrically nonlinear designs of the U-shaped plate. It can be noted that the application of RBTO shows different resulted optimized shapes relative to those which are obtained in case of deterministic designs.

**Table 6:**
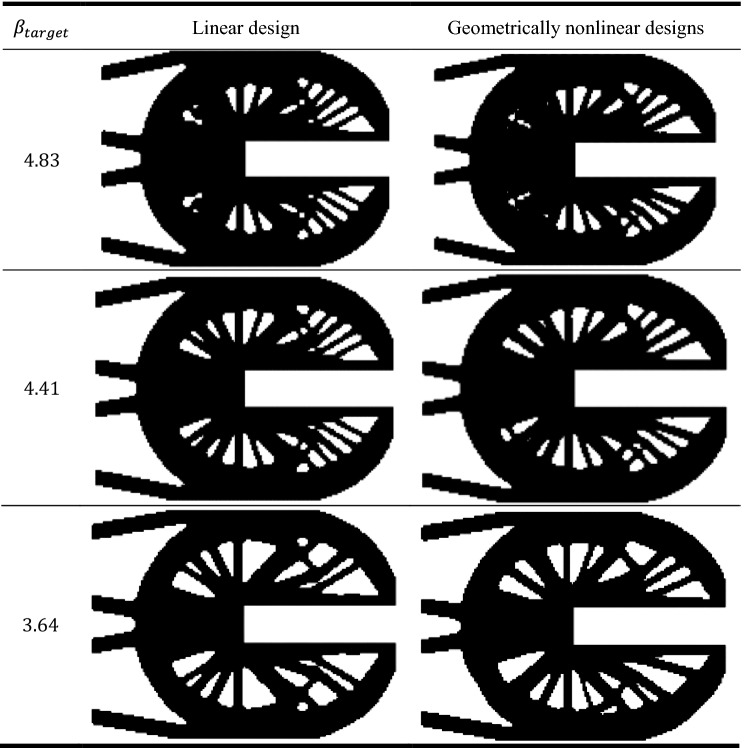
Topological resulted shapes of the U-shaped plate in case of probabilistic design.

**Figure 7:**
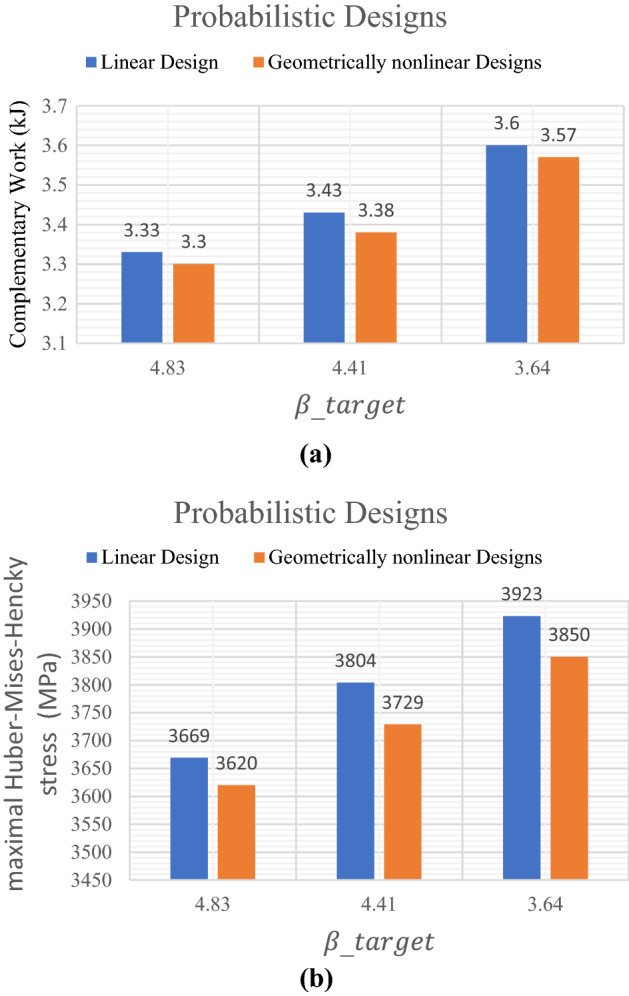
Comparison between linear and geometrically nonlinear probabilistic design by considering: (**a**) complementary work, (**b**) maximal Huber-Mises-Hencky stress.

Similar to the comparisons of the deterministic results, here a new comparison between results of probabilistic linear and geometrically nonlinear topology optimization designs are shown in Fig. 7. It can be noted that the complementary work value for linear design is smaller than that for geometrically nonlinear design for the same value of ${\beta }_{target}$. It can be also noticed from Fig. 7.a that for ${\beta }_{target}=4.83$, the complementary work has been decreased by $1.00\%$ from $3.33$ kJ in case of linear design to $3.30$ kJ in case of geometrically nonlinear design. in case of ${\beta }_{target}=3.64$, the complementary work has been reduced by $0.83\%$ from $3.60$ kJ to $3.57$ kJ.

It can be seen from Fig. 7b, the maximal Huber-Mises-Hencky stress has been decreased by $1.36\%$ from $3669$ MPa in case of linear design to $3620$ MPa in case of geometrically nonlinear design when ${\beta }_{target}=4.83$. While for the ${\beta }_{target}=3.64$, the maximal Huber-Mises-Hencky stress has been reduced by $1.86\%$ from $3923$ MPa to $3850$ MPa.

Figure 7a shows that in case of linear and geometrically nonlinear, as ${\beta }_{target}$ decreases the complementary work increases too for both topology optimization designs. Besides, Fig. 7b, indicates that also maximal Huber-Mises-Hencky stress as ${\beta }_{target}$ decreases in case of probabilistic linear and geometrically nonlinear topology optimization.

The results of complementary work and maximal Huber-Mises-Hencky stress have been efficiently reduced form those results which have been obtained from deterministic designs when introducing ${\beta }_{target}$ constraint for both linear and geometrically nonlinear elastic design.

### 2D elasto-plastic problem: U-shaped plate

Probabilistic elasto-plastic geometrically nonlinear topology optimization of 2D U-shaped plate problem is considered as third example in this study. Also, Monte-Carlo technique is considered. For reliability assessment, ${V}_{f}$ is considered random. Boundary conditions, the number of Monte-Carlo simulations and material properties of this example are considered the same as what considered in the example in section ($6.2$). BESO parameters are $ER=1.75\%$, ${AR}_{max }=1\%$, ${r}_{min }= 0.006$ m and $\tau =1\%$. The yield stress equals ${\upsigma }_{y}=135$ MPa. The plastic-limit load multiplier is assumed ${m}_{p}=3.18$, the initial predefined load is equal to ${F}_{0}=4$ kN and the ultimate load is ${F}_{\mathrm{ult}}=15.8$ kN. $60\% and 5\%$ are considered as the mean value and the variance, respectively of the random variable ${V}_{f}$. To illustrate the action of load multiplier, four load cases are introduced: ${F}_{1}= {0.9 F}_{0}$, ${F}_{2}= {1.125 F}_{0}$ , ${F}_{3}= {1.875 F}_{0} ,{F}_{4}= {3.125 F}_{0}$.

Table 7 represents the Huber-v. Mises-Hencky $(HMH$) stresses of optimum topology designs of probabilistic geometrically nonlinear analysis for the three different ${\beta }_{target}$ values. Considering that the value of load multiplier ${F}_{1}= {0.9 F}_{0}$. Also, Tables 8, 9 and 10 represent the same comparison between the obtained results for ${F}_{2}= {1.125 F}_{0}$ , ${F}_{3}= {1.875 F}_{0} ,{F}_{4}= {3.125 F}_{0}$, respectively.

According to the obtained results, we can say that in case of lowest load multiplier ($${F}_{1}= {0.9 F}_{0}),$$ the mean stress has been increased by $$1.93\%$$ from $$179.47$$ MPa when $${\beta }_{target}=4.83$$ to $$183$$ MPa when $${\beta }_{target}=3.64.$$ while in case of highest load multiplier ($${F}_{4}= {3.125 F}_{0}$$), the mean stress has been increased by $$2.34\%$$ from $$219.74$$ MPa when $${\beta }_{target}=4.83$$ to $$225 MPa$$ when $${\beta }_{target}=3.64.$$

The obtained results indicate that for the first case of load multiplier which is the lowest acting load case, there is barely existence of the plastic zones. On the other hand, for the highest acting load case, plastic zones are obtained largely. Besides, it can be seen that for each case of load multiplier, the mean stress increases as ${\beta }_{target}$ decreases. Thus, this example validates the efficiency of the new approach which extended BESO to consider reliability-based elasto-plastic geometrically nonlinear topology optimization.

Table 11 represents a comparison between the results of the topology optimization of linear, geometrically nonlinear, and elasto-plastic models based on the resulted optimized shapes and the complementary work by considering the magnitude of the applied load $12.5$ kN and ${\beta }_{target}=3.64$. Taking into consideration that for elasto-plastic model, the stress intensity scale can be seen referring to Table 10. It can be noticed from Table 11 that each model has different resulted optimized shape. Also, the obtained complementary work values are different too. The complementary work value for elasto-plastic model is higher than that for linear and geometrically nonlinear models, thus it seems that the elasto-plastic model can improve the control for maximal stress of the structures while it deteriorates the structural stiffness.

Finally, it is worth mentioning that a personal computer with intel® core™ i7-7700HQ @ 2.80 GHz processor and 16.0 GB RAM is used to perform the topology optimization. And due to the importance of time-consuming criteria in topology optimization, the CPU time for the proposed method of reliability-based topology optimization of each model is given in Table 12.

**Table 7:**
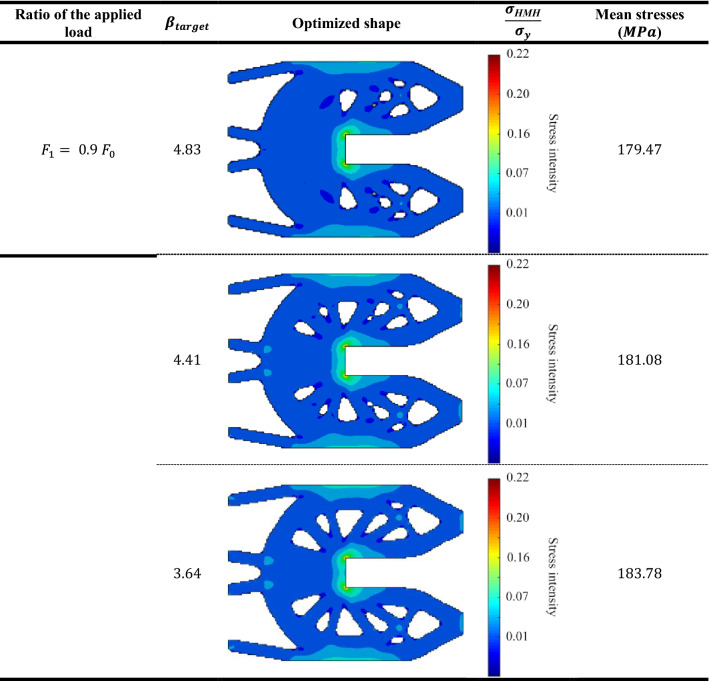
$HMH$ stresses and optimum topologies according to various load multiplier.

**Table 8:**
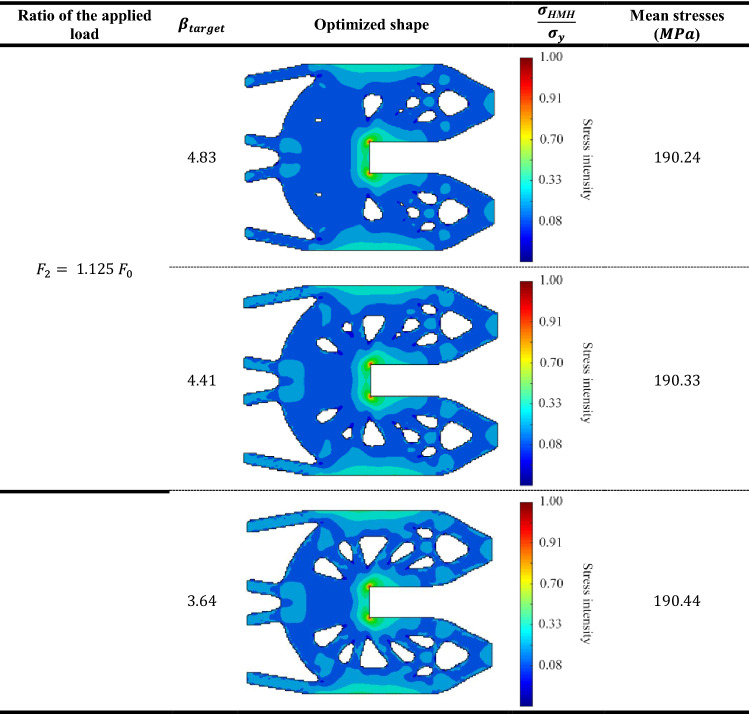
$HMH$ stresses and optimum topologies according to various load multiplier.

**Table 9:**
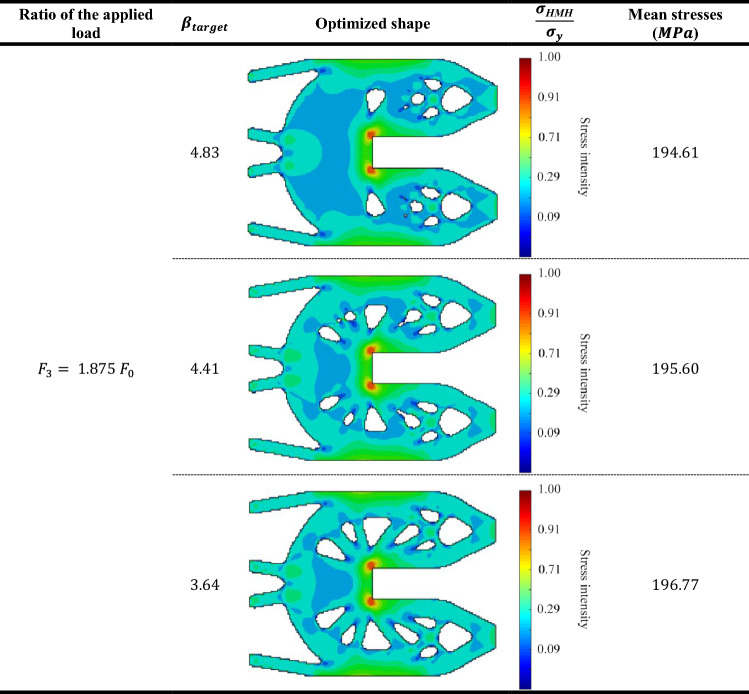
$\mathrm{HMH}$ stresses and optimum topologies according to various load multiplier.

**Table 10:**
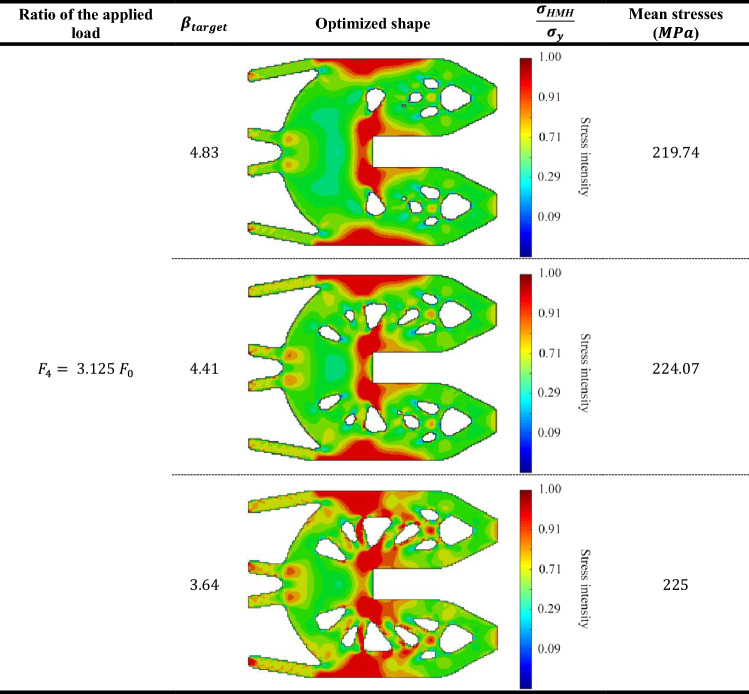
$HMH$ stresses and optimum topologies according to various load multiplier.

**Table 11:**
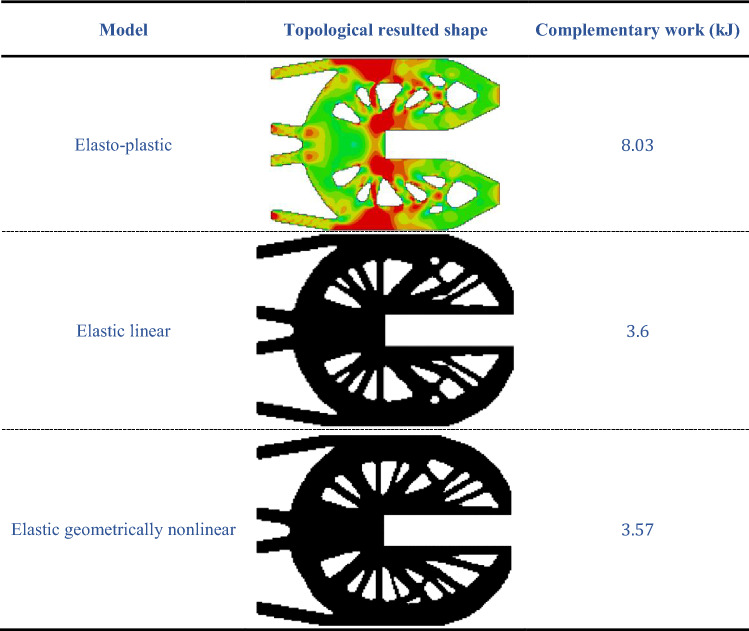
Design results and complementary work values of the U-shape models.

**Table 12:** The computational time of the proposed method.

TO problem	CPU time for the whole optimization process (second)
Linear model	5160
Geometrically nonlinear model	5400
Elasto-plastic model	5820

## Conclusions

The fundamental commitment of this paper is to extend the topology optimization geometrically nonlinear elastic and elasto-plastic structures in the existence of uncertainties by adopting the prolonged BESO method to achieve reliability-based design. For this reason, because of uncertainties, volume fraction ${V}_{f}$ is considered as random variable throughout optimization process. To be specific, structural topology optimization is combined with the reliability theory. Thus, finding the optimal topology solution of structures considering satisfying the reliability constraint. Furthermore, the advanced Monte-Carlo simulation technique is adjusted to analyze the reliability through probability of failure calculation, thus the reliability index. Plastic-limit load multiplier method is considered to solve elasto-plastic topology optimization problems. The proposed structural topology optimization method can be deliberated as an efficient tool for facing considerable designing problems related to the minimization of the mean compliance of elasto-plastic structures which are capable to carry given forces.

The solutions of the benchmark problem obviously demonstrate the value and practicality of the proposed method. In addition, the topology optimization results of the other considered problems indicate that there is a serious relationship between reliability-level and the objective function, with the results compatible to our knowledge. Consequently, in executing reliability-based design, performance and safety must be considered.

The proposed work can be summed up into the following key conclusions:

- Since the failure’s parameters are introduced for probabilistic designs, the values of complementary work in case of geometrically nonlinear designs are lower than which are obtained in deterministic designs.
- By considering multiple values of reliability index for probabilistic design, the complementary work and the maximal Huber-Mises-Hencky stress increases as the reliability index decreases.
- When defining a probabilistic constraint on volume fraction, the final optimum topological shape of the structures is changed from the deterministic design.
- The obtained maximal Huber-Mises-Hencky stresses values in case of elastic geometrically nonlinear design is lower than the obtained values for linear design in deterministic design. On the other hand, for probabilistic design, it can be noticed that as the reliability index decreases the maximal stress increases too for linear designs and geometrically nonlinear designs as well.
- By considering multiple values of volume fraction for deterministic designs, it can be noticed that the complementary work and the maximal Huber-Mises-Hencky stress increase as volume fraction decreases.
- In case of elasto-plastic design, the first case of load multiplier which is the lowest acting load case, there is barely existence of the plastic zones. On the other hand, for the highest acting load case, plastic zones are obtained largely.

The work presented in this study can be deemed as significant improvement towards a more reasonable and broader framework for elastic and elasto-plastic topological designs of geometrically nonlinear analysis by considering plastic-limit analysis under reliability constraint. Regardless, additional research is anticipated to include other nonlinear problems.

## Data Availability

The datasets generated during and/or analyzed during the current study are available in the main manuscript.
